# Human Hemoglobin-Based Zinc–Air Battery in a Neutral Electrolyte

**DOI:** 10.1021/acs.energyfuels.3c02513

**Published:** 2023-09-25

**Authors:** Valentín García-Caballero, Sebastián Lorca, Marta Villa-Moreno, Álvaro Caballero, Juan J. Giner-Casares, Antonio J. Fernández-Romero, Manuel Cano

**Affiliations:** † Departamento de Química Física y Termodinámica Aplicada, Instituto Químico para la Energía y el Medioambiente, Universidad de Córdoba, E-14014 Córdoba, Spain; ‡ Grupo de Materiales Avanzados para la Producción y Almacenamiento de Energía, Universidad Politécnica de Cartagena, Aulario II, Campus de Alfonso XIII, 30203 Cartagena, Spain; § Departamento de Química Inorgánica e Ingeniería Química, Instituto Químico para la Energía y el Medioambiente, Facultad de Ciencias, Universidad de Córdoba, E-14014 Córdoba, Spain

## Abstract

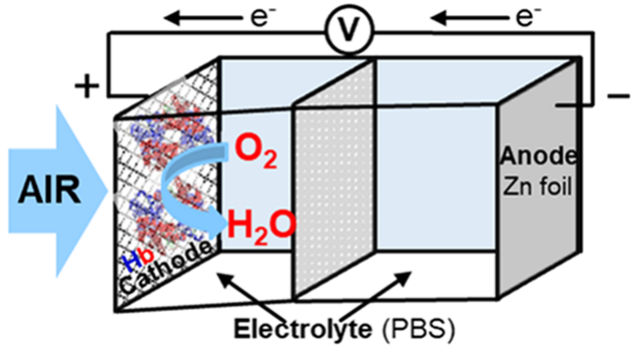

The use of human hemoglobin (Hb) as a catalytic component of the air electrode in a primary zinc–air battery with a neutral electrolyte has been investigated. Three different electrode modifications, using the drop-casting method, with Hb and Nafion were first tested in a three-electrode cell, obtaining the best oxygen electroreduction (ORR) performance and long-term stability with a Hb plus Nafion (Hb–Nafion)-modified electrode. The latter Hb–Nafion-based air electrode provided a higher specific capacity and discharge time than the opposite order (Nafion–Hb).

## 1 Introduction

Portable devices as well as energy demand have increased exponentially in recent years, requiring the development of low-cost and sustainable energy conversion and storage systems.^1^ Among these are batteries, which are chemical devices that store electrical energy in the form of chemicals, and through reversible electrochemical reactions, they convert the stored chemical energy into direct current electric energy.^2^ Currently, Li-ion batteries are the most applied systems in the automotive and portable device sector. However, several drawbacks make it necessary to search for other types of batteries based on more abundant, safer, and less expensive materials.^3^

Nowadays, aqueous Zn–air batteries (ZABs) are emerging as promising candidates for their safety, low cost, eco-friendliness, and high theoretical capacity.^4−6^ Although most of the reported studies on ZABs use alkaline electrolytes as a result of their good conductivity, a relevant issue is the limited durability associated with the hydrogen evolution reaction, dendrite, and carbonate formations.^7,8^ Recently, neutral and near-neutral electrolytes have been proposed to overcome these limitations.^9−12^

In addition, nowadays, there is a great interest for the development of biocompatible batteries to be applied in electronic medical implants or point-of-care monitoring systems.^13^ Herein, for the first time ever, the setup of a primary Zn–air battery with a neutral electrolyte, containing human hemoglobin (Hb) as an electrocatalyst for the oxygen reduction reaction (ORR), is proposed. Hb is a well-known iron-containing oxygen transport protein, which is present in erythrocytes of almost all vertebrates.^14^ Basically, each Hb protein contains four hemo groups [i.e., iron(II) metalloporphyrin] that act as a single-atom catalyst (SAC), which are protected by surrounding protein structures against any potential catalyst poisoning.^15^ Previously, Compton and co-workers demonstrated the successful electrocatalyst performance for the ORR of Hb modifying a glassy carbon electrode (GCE) using a porous Nafion layer and phosphate-buffered saline (PBS, pH 7.4) as the electrolyte.^15^ Although the ORR is a reaction involved in the cathode of both metal–air batteries and fuel cells, Compton and co-workers did not apply these Hb redox properties in any device. We consider that this study paves the way for the development a biological-based ZAB, which could be useful for the future development of a body-integrated self-powered system for wearable and implantable applications.^16,17^

## 2 Materials and Methods

### 2.1 Material Preparation

Human Hb was purchased from Sigma-Aldrich, which was diluted in Milli-Q water, with a final concentration of 5 mg mL^–1^. Nafion (5 wt %) in lower aliphatic alcohols and water (containing 15–20% water) was purchased from Sigma-Aldrich. Three different modifications with Hb were performed using the sequential drop-casting method on the working electrode surface, such as Nafion–Hb, Nafion–Hb–Nafion, and Hb–Nafion (Figure S1 of the Supporting Information). The electrode modification through the sandwich structure (Nafion–Hb–Nafion) was performed using the same volumes used by Compton and co-workers.^15^ For the other combinations (Nafion–Hb and Hb–Nafion), 10 μL of Hb and 2 μL of Nafion were used to modify a GCE of 3 mm in diameter. For the air or cathode electrode in ZABs, 33 μL of Hb and 6.6 μL of Nafion were used to modify the carbon cloth gas diffusion layer (GDL, from the fuel cell) electrode of 10 mm in diameter. The final mass loading was 50 μg of Nafion and 0.165 mg of Hb. A 0.3 M PBS solution at pH 7.4 was used as the electrolyte.

### 2.2 Electrochemical Measurements

A PalmSens4 potentiostat/galvanostat from PalmSens BV was used for electrochemical analyses in a standard electrochemical cell from BASi research products (with a 15 mL glass cell). Ag/AgCl, a graphite rod, and a GCE were used as reference, counter, and working electrodes, respectively. A rotating disk-ring electrode (RRDE) system (RRDE-3A from Als Co., Ltd., Tokyo, Japan) with a GC disk and an Au ring [using a constant potential of 1.4 V versus reversible hydrogen electrode (RHE)] was employed for the kinetic analysis. For the ZABs, a BioLogic BCS-810 battery cycler was used to perform galvanostatic discharge analysis. The specific capacity was calculated dividing the capacity (mAh) value obtained for each battery against the catalyst mass loaded on the cathode.

### 2.3 Material Characterization

A XPS SPECS PHOIBOS150 MCD spectrometer was used for X-ray photoelectron spectroscopy (XPS) studies (X-ray source with Mg and Al anodes, Al and Ag monochromatic source, and 1253.6 eV). Spectra were recorded in constant energy mode at 30 eV and using a 720 μm diameter analysis area. CasaXPS, version 2.3.16, software package was used for data analysis. The process of measuring morphology and elemental composition was carried out using field emission scanning electron microscopy (FESEM) ZeissCrossbeam 350 (Carl Zeiss Microscopy GmbH, Germany). The scanning electron microscopy (SEM) observation conditions were 10 kV acceleration voltage and 5 mm working distance. The image was generated with the secondary electron signal (SE secondary electron), and the SmartSEM 6.07 software from Zeiss was used for its acquisition. For energy-dispersive X-ray spectroscopy (EDX), an energy-dispersive X-ray detector at 10 kV acceleration voltage, from Oxford Instrument, U.K., and AZtec software, version 5.0.7577.2, was employed.

## 3 Results and Discussion

### 3.1 Effect of Nafion Modification

**Figure 1:**
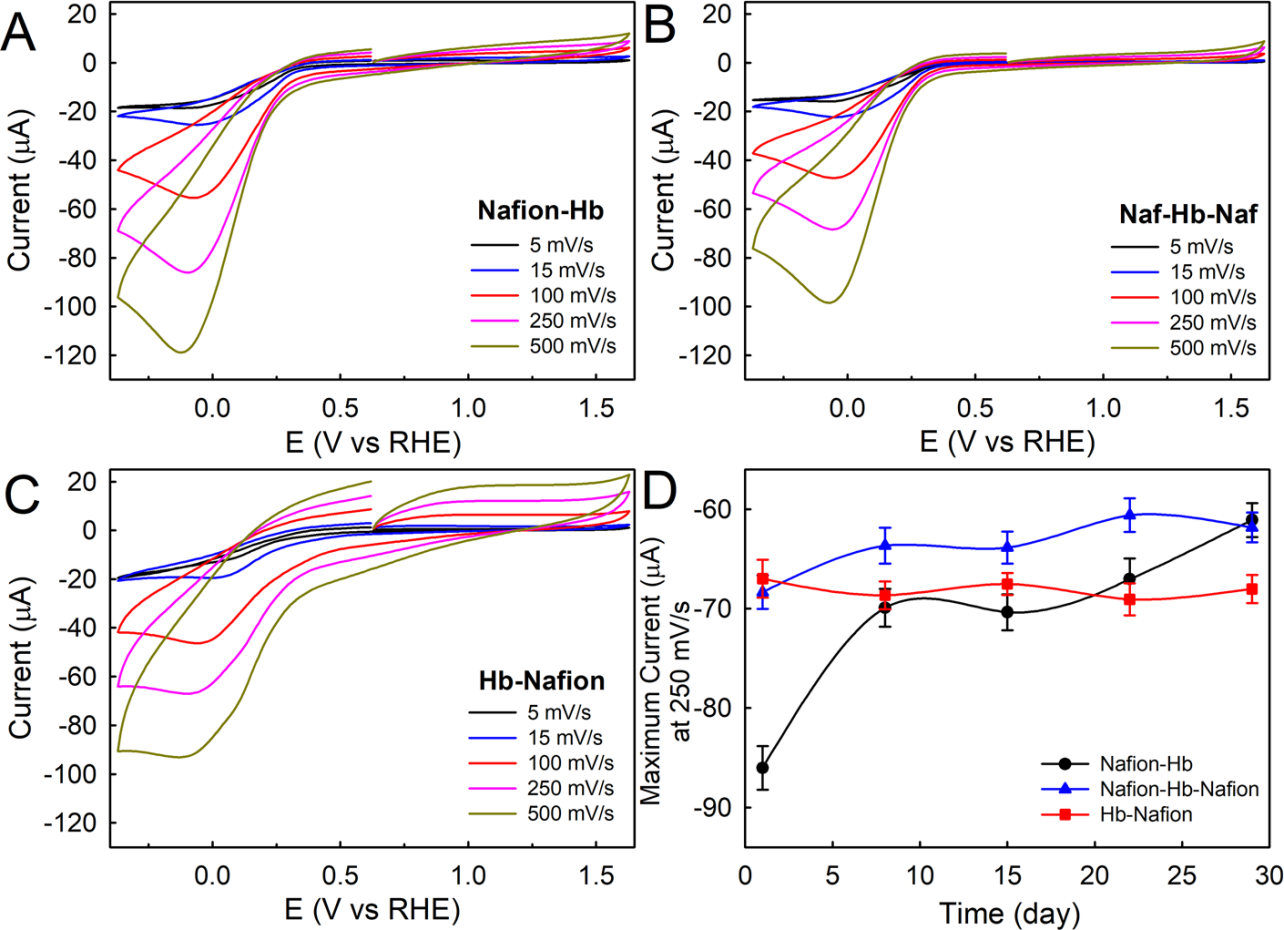
Representative CV curves of day 1 for GCE modified with (A) Nafion–Hb, (B) Nafion–Hb–Nafion, and (C) Hb–Nafion at different scan rates in O_2_-saturated 0.1 M PBS (pH 7.4) and (D) comparative durability test for 1 month at 250 mV/s scan rate.

As previously reported by Compton and co-workers,^15^ a Nafion ionomer was used to immobilize Hb on the electrode surface. First, the effect of the electrode modification was investigated. Panels A–C of Figure 1 show the resulting cyclic voltammetry (CV) for three different electrode modifications, such as Hb–Nafion, Nafion–Hb–Nafion, and Nafion–Hb, at several scan rates in oxygen-saturated PBS buffer (pH 7.4). A characteristic peak of oxygen electroreduction at 0.0 versus RHE can be observed in these three samples analyzed, which is consistent with previous reports.^15,18^ It should be noted that Nafion–Hb and Hb–Nafion provided the maximum current and lower onset potential values, respectively. Moreover, the latter electrode modification exhibited the highest double-layer capacitance (*C*_dl_) in the non-faradaic region, indicating highly exposed active sites and, consequently, larger potential electrocatalytic activity for the ORR.^19^ The analysis of the electrochemical surface area (ECSA) for bare GCE and the three different electrode modifications with Hb and Nafion was also carried out. The used method is based on the measurement of the differential capacitance in the electrical double-layer region by applying the Gouy–Chapman theory.^19^Figure S5 of the Supporting Information shows the resulting CV curves in the non-Faradaic region for the different samples at five different scan rates (from 20 to 100 mV s^–1^) in nitrogen-saturated 0.1 M PBS, demonstrating the near-rectangular shape of the CV curves and confirming the non-faradaic electrical double-layer (EDL) charging capacitive behavior. Figure S6A of the Supporting Information compares CV curves obtained in the non-faradaic region for the different samples at 60 mV s^–1^, while Figure S6B of the Supporting Information plots the difference in current density between positive and negative potential cycles (Δ*J* = *J*_anodic_ – *J*_cathodic_) against different scan rates obtained through Figure S5 of the Supporting Information. Then, the double-layer capacitances of *C*_dl_ can be calculated using the equation: *C*_dl_ = Δ*J*/2ν, obtaining values of 19.9, 25.6, 22.2, and 18.3 μF cm^–2^ for bare GCE and GCE modified with Hb–Nafion, Nafion–Hb, and Nafion–Hb–Nafion, respectively. These results clearly demonstrate that GCE modified with Hb–Nafion exhibits the highest ECSA value, while the sandwich modification exhibits the worst value.

In addition, the long-term stability was checked for 1 month, storing the modified electrodes at 4 °C and immersing in the same PBS solution used as the electrolyte. Figure 1D compares the variation with time of the maximum current at the scan rate of 250 mV/s for the three different electrode modifications employed (Figure 1D summarizes the results shown in Figures S2–S4 of the Supporting Information). The combination of Hb plus Nafion (Hb–Nafion) keeps the current along the time course almost constant, therefore displaying the best stability behavior, followed by Nafion–Hb–Nafion. The worst durability was clearly observed with the Nafion–Hb sample, reducing the current from −86 to −61 μA (i.e., a loss of 29%). This fact can be attributed to the high solubility of Hb in water as well as the lack of protection with Nafion, causing its loss over time toward the electrolyte.

The ORR kinetics were compared for Nafion–Hb and Hb–Nafion using a RRDE (Figure S7 of the Supporting Information). The limited diffusion current densities of both combinations linearly increased with increasing rotation rates, revealing a typical diffusion-controlled electrochemical process.^19^ As observed in Figure S8 of the Supporting Information obtained through Figure S7 of the Supporting Information using eqs S1 and S2 of the Supporting Information,^20,21^ the electrode modified with Hb–Nafion displayed a better kinetic reaction than that with Nafion–Hb, obtaining for Hb–Nafion a dominant four-electron pathway and a lower percentage of HO_2_^–^ than for Nafion–Hb.

**Figure 2:**
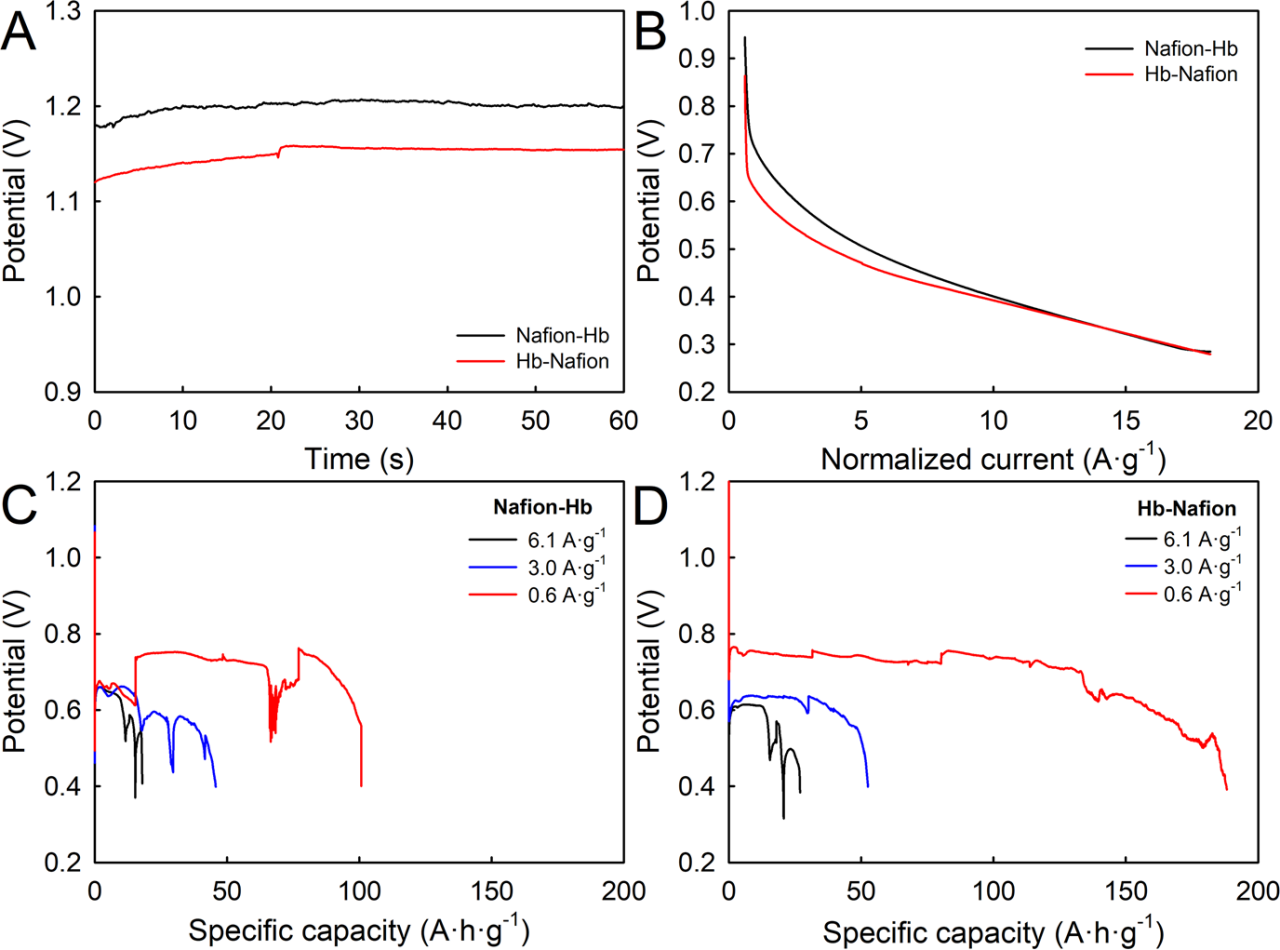
(A) OCP and (B) linear sweep potentiometry (LSP) for GDL modified with Nafion–Hb and Hb–Nafion and (C and D) galvanostatic discharge curves of a Zn/0.3 M PBS/air battery using GDL modified with (C) Nafion–Hb or (D) Hb–Nafion as air electrodes, respectively.

Next, their potential application in real devices, such as ZABs, was investigated. For this, two combinations, Nafion–Hb (the worst combination) and Hb–Nafion (the best combination), supported on GDL were used as the air electrode in a flooded ZAB, containing a Zn plate anode and a 0.3 M PBS (pH 7.4) electrolyte.^7^Figure 2A compares the resulting chronopotentiometric potential–time curves under open circuit potential (OCP) conditions at an air atmosphere and room temperature. OCP defines the nature of the charges developed at the electrode–surface interface with no current flows. Nafion–Hb exhibited an OCP potential of ca. 1.20 V, while Hb–Nafion displayed a value of ca. 1.15 V. These findings suggest that both interfaces become positively charged and without a significant difference. The higher resulting OCP value of Nafion–Hb may be a consequence of an improved quality of the electric conductivity of this electrode, which could indicate a relatively lower kinetic barrier. Figure 2B shows the voltage versus current density plots for both ZABs, which were recorded basically to choose the appropriate discharge intensities at which a relatively high potential is obtained. Both curves had a very similar shape, choosing 0.6, 3, and 6.1 A g^–1^ as discharge intensities. These values were the same proposed previously by Park and co-workers, measuring a ZAB-based on an iron acetylacetonate complex.^4^ Panels C and D of Figure 2 show galvanostatic discharge curves at the previous chosen intensities for the Nafion–Hb ZAB and Hb–Nafion ZAB, respectively. It was worth noting that, for all of the intensities tested, the resulting specific capacities of the Hb–Nafion ZAB were significatively higher than those for the Nafion–Hb ZAB, which could be attributed to the higher diffusion of Hb into the electrolyte solution in the latter case (i.e., Hb was directly exposed to the PBS solution without any protection of the Nafion layer). Table 1 summarizes the resulting specific capacities (with respect to the catalyst mass) and the discharge times at 0.6, 3, and 6.1 A g^–1^ for both electrode modifications tested in ZABs. As expected, Hb–Nafion provided the better features as an electrocatalyst for these cells. Furthermore, these results greatly exceed the discharge times reported previously for other ZABs based on an iron acetylacetonate complex.^4^ To the best of our knowledge, no reference was found in the literature reporting a ZAB based on a biological molecule, which is directly used without any chemical decomposition and/or thermal treatment.

**Table 1:** Summary of the ZAB Characteristics Obtained with Both Air Electrodes

	Nafion–Hb		Hb–Nafion	
applied current (A g^–1^)	A h g^–1^	hours	A h g^–1^	hours
6.1	17.9	2.95	26.9	4.44
3.0	45.7	15.10	52.6	17.36
0.6	100.8	166.30	188.2	310.50

**Figure 3:**
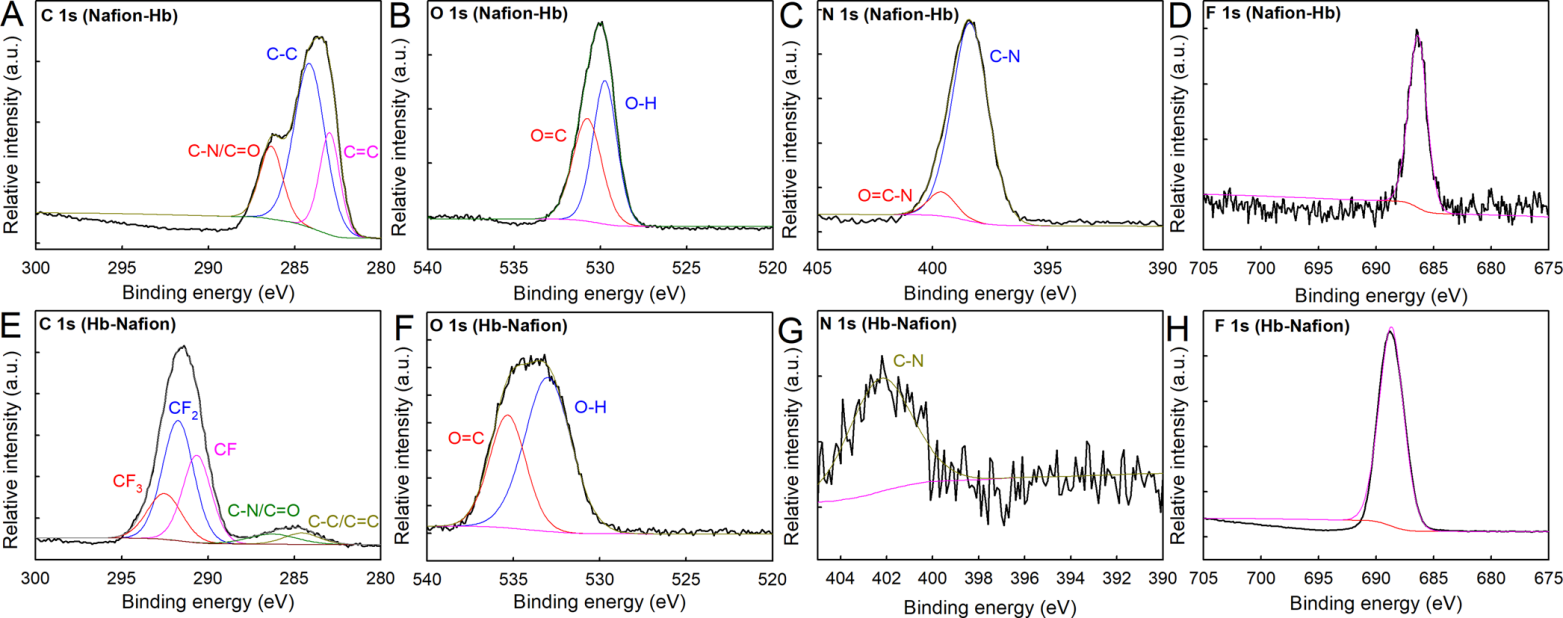
HR XPS spectra of (A and E) C 1s, (B and F) O 1s, (C and G) N 1s, and (D and H) F 1s for GDL modified with Nafion–Hb and Hb–Nafion, respectively.

Afterward, XPS analysis was carried out to examine the chemical composition on the surface of the two different air electrodes used in ZABs. Overall, Figure 3 shows a significantly positive binding energy (BE) peak shift for GDL modified with Hb–Nafion that is associated with the greater amount of fluoride exposed to the surface (i.e., highly electronegative element).^22^Figure 3A shows the high-resolution (HR) XPS spectra of the C 1s region for GDL modified with Nafion–Hb, where three different peaks at 286.37, 284.15, and 282.98 eV associated with C–N/C=O, C–C, and C=C groups, respectively, can be observed.^23^Figure 3E for Hb–Nafion shows the C 1s region for the Hb–Nafion modification, highlighting three additional peaks associated with the presence of Nafion (polymeric fluoride compound). These peaks at 292,53, 291.74, and 290.65 eV are associated with CF_3_, CF_2_, and CF, respectively.^24^Figure 3B shows the HR XPS spectra of the O 1s region for GDL modified with Nafion–Hb, where two peaks at 530.76 and 529.73 eV attributed to O=C and O–H groups, respectively, can be identified.^23^Figure 3F for Hb–Nafion shows the same peaks but widened and shifted to higher binding energies (at 535.34 and 533.01 eV), as commented above for the presence of fluoride. Figure 3C shows the HR XPS spectra of the N 1s region for GDL modified with Nafion–Hb modification, where two different peaks that appear at 399.62 and 398.37 eV associated with O=C–N and C–N groups, respectively, can be observed.^23^Figure 3G for Hb–Nafion shows only a shifted peak at 402.26 eV associated with the C–N group. Last, Figure 3D shows the HR XPS spectra of the F 1s region for GDL modified with Nafion–Hb, where a characteristic peak of fluoride at 686.38 eV can be observed.^24^ Similar BE displacement previously described can be observed in Figure 3H for the Hb–Nafion modification. All of these results demonstrate that Hb–Nafion modification provides not only the better ZAB features but also the more efficient Nafion coating, which prevents Hb diffusion into the electrolyte and, thus, favors longer discharge capacities. Although HR XPS spectra of the Fe 2p region were recorded for both GDL modifications, unfortunately, iron could not be detected probably as a result of the low iron concentration in both samples (Figure S9 of the Supporting Information). Survey XPS spectra have also been included in Figure S10 of the Supporting Information.

Finally, SEM and its corresponding EDX element mapping analysis is provided for GDL modified with Nafion–Hb (Figures S11 and S12 of the Supporting Information) and Hb–Nafion (Figures S13 and S14 of the Supporting Information). Overall, SEM images of both samples show similar rough and homogeneous surface morphologies, although a large crack is observed only for Hb–Nafion modification. The obtained elemental compositions from EDX analysis matched well with those obtained by XPS analysis for these two samples. The element mapping results for GDL modified with Nafion–Hb indicates the existence of elements C, N, O, F, Fe, and S (in descending order, as shown in Figure S12G of the Supporting Information), confirming that the Hb protein is on Nafion, basically as a result of the amount of N associated with the Hb protein being higher than the amount of F from the Nafion polymer (Figure S11 of the Supporting Information). In addition, a uniform distribution of C, N, O, F, Fe, and S atoms can be observed throughout the entire GDL surface (panels A–F of Figure S12 of the Supporting Information). The element mapping results for GDL modified with Hb–Nafion indicate the existence of elements C, F, O, N, S, and Cl (in descending order, as shown in Figure S14H of the Supporting Information), confirming that Nafion is on Hb basically as a result of the majority amount of F on the surface, which is associated with Nafion (Figure S13 of the Supporting Information). In addition, a uniform distribution of C, F, O, N, S, Cl, and Fe atoms could be observed throughout the entire GDL surface (panels A–F of Figure S14 of the Supporting Information). It should be noted that Nafion contains a trace amount of chlorine.^25^

## 4 Conclusion

The successful application of human Hb as an air electrode in a ZAB, achieving significant specific capacity and discharge time values, has been demonstrated for the first time ever. In addition, two different electrode modifications were characterized by XPS, FESEM, and electrochemical analysis, obtaining better features (in terms of available active sites and durability) with the Hb–Nafion combination as a result of Nafion coating that was essential for Hb stabilization in the air electrode.

## Supporting Information Available

Schematic representation, CV curves at different scan rates of the long-term stability studies, RRDE analysis for the ORR to calculate the electron transfer number, ECSA analysis for the three different GCE modifications, survey XPS spectra, HR XPS of Fe 2p spectra, and SEM–EDX analysis of GDL modified with Nafion–Hb and Hb–Nafion (PDF)
