# Supplementary material for: Human Hemoglobin-Based Zinc–Air Battery in a Neutral Electrolyte
Source: Energy Fuels. 2023 Sep 25;37(23):18210–5. doi: 10.1021/acs.energyfuels.3c02513 (PMC10714475; doi:10.1021/acs.energyfuels.3c02513)
Supplement: Supplementary file 1 — ef3c02513_si_001.pdf [file ef3c02513_si_001.pdf]

Supporting Information

A Human Hemoglobin-based Zinc-Air Battery in Neutral Electrolyte

Valentín García-Caballero,[†] Sebastián Lorca,[‡] Marta Villa-Moreno,[†] Álvaro Caballero,[□] Juan J. Giner-Casares,[†] Antonio J. Fernández-Romero[†,‡,*] and Manuel Cano[†,*]

[†]Departamento de Química Física y Termodinámica Aplicada, Instituto Químico para la Energía y el Medioambiente, Universidad de Córdoba, E-14014 Córdoba, Spain.

[‡]Grupo de Materiales Avanzados para la Producción y Almacenamiento de Energía, Universidad Politécnica de Cartagena, Aulario II, Campus de Alfonso XIII, 30203 Cartagena, Spain.

[□]Departamento de Química Inorgánica e Ingeniería Química, Instituto Químico para la Energía y el Medioambiente, Universidad de Córdoba, E-14014 Córdoba, Spain.

*E-mail corresponding authors: antonioJ.fernandez@upct.es; q82calum@uco.es

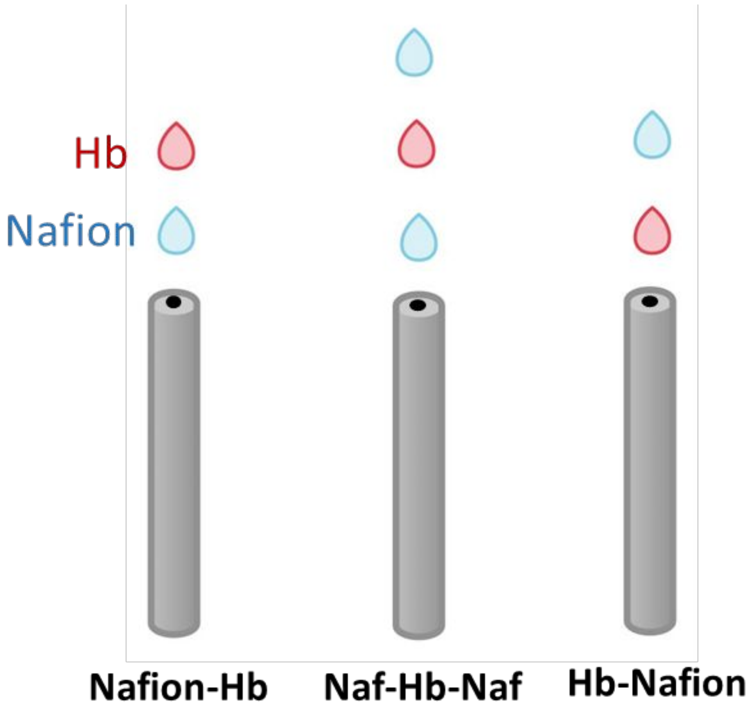

Figure S1. Schematic representation of the three different electrode modifications with Hb performed using sequential drop-casting method on working electrode surface, such as: Nafion-Hb, Nafion-Hb-Nafion and Hb-Nafion.

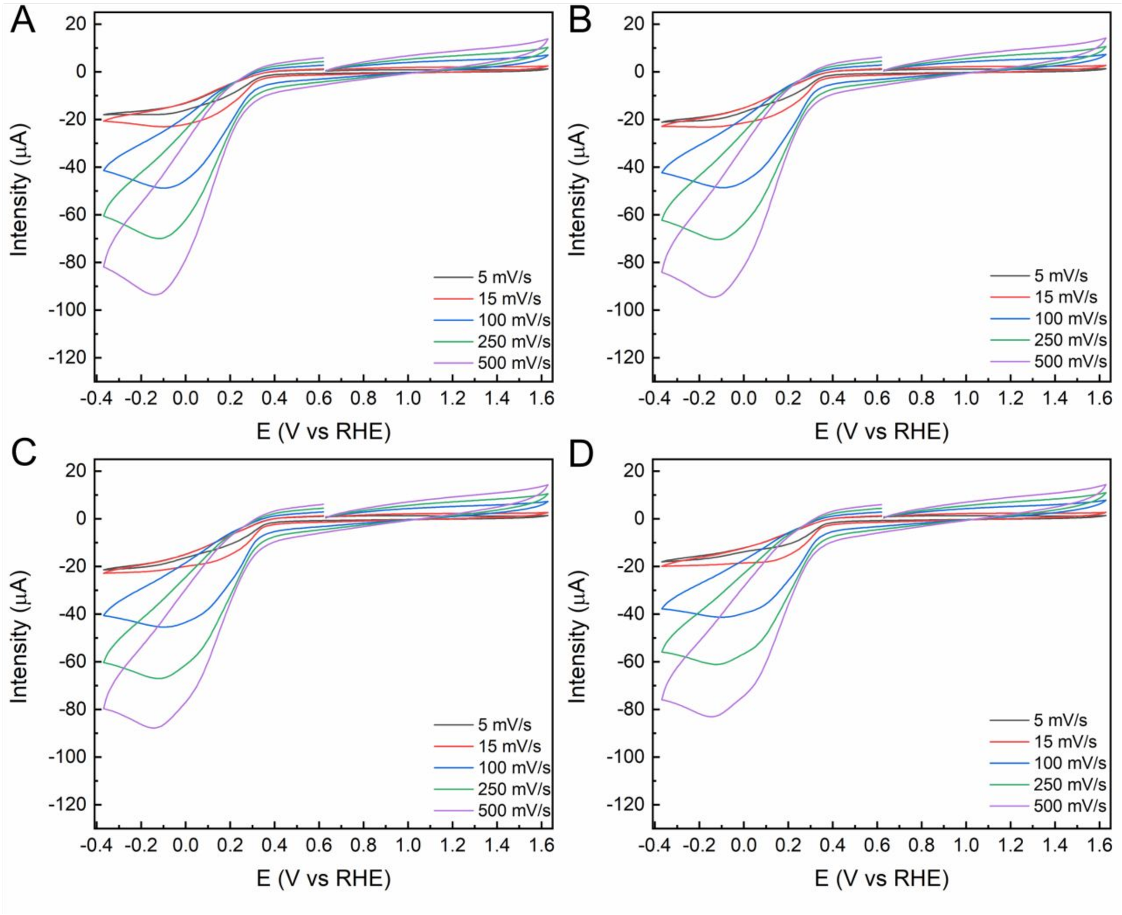

Figure S2. Representative cyclic voltametric (CV) curves obtained at different scan rates for the GCE modified with Nafion-Hb in O_2-saturated 0.1 M PBS after 8 days (A), 15 days (B), 22 days (C) and 29 days (D).

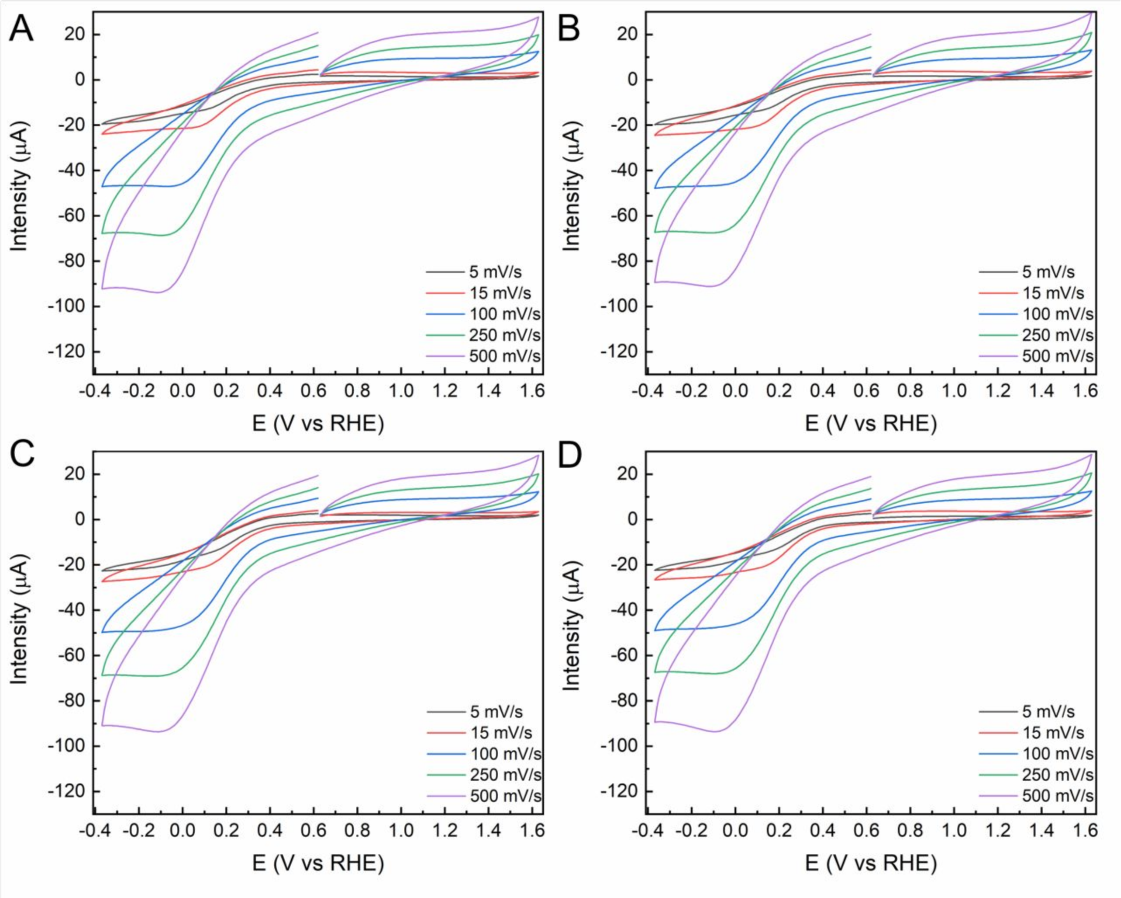

Figure S3. Representative CV curves obtained at different scan rates for the GCE modified with Hb-Nafion in O_2-saturated 0.1 M PBS after 8 days (A), 15 days (B), 22 days (C) and 29 days (D).

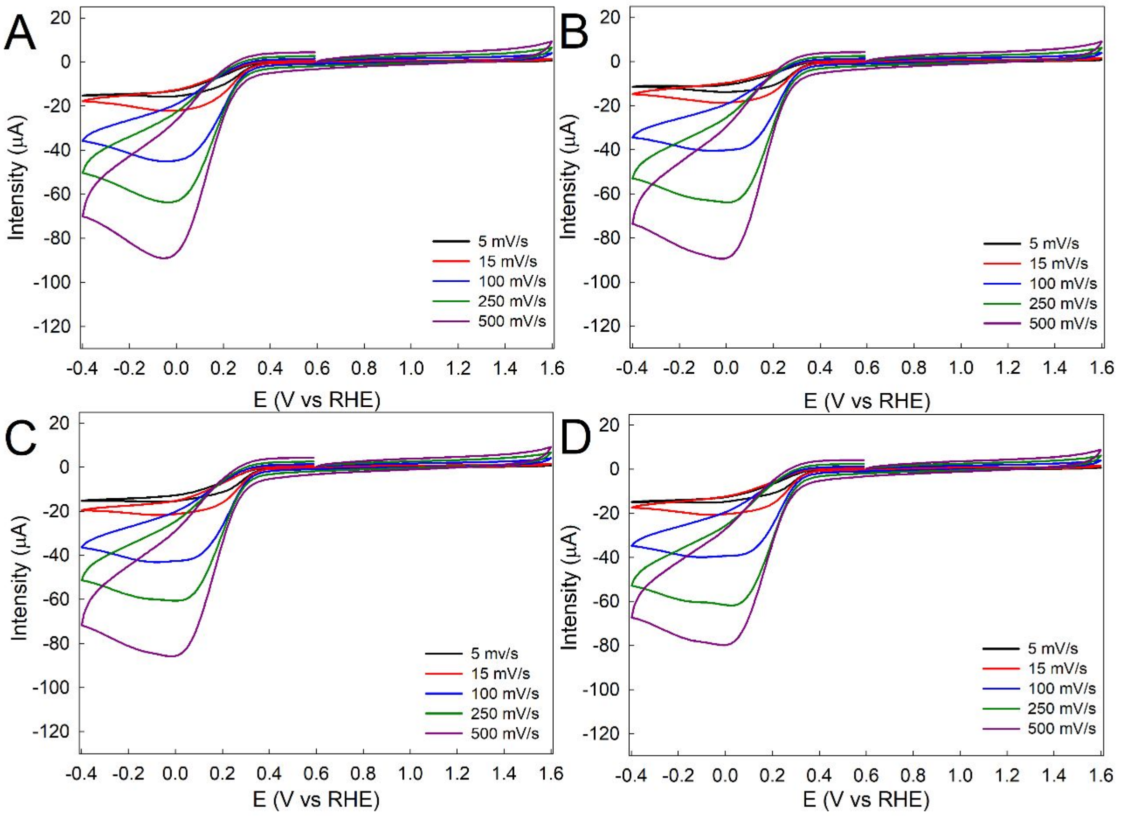

Figure S4. Representative CV curves obtained at different scan rates for the GCE modified with Nafion-Hb-Nafion in O_2-saturated 0.1 M PBS after 8 days (A), 15 days (B), 22 days (C) and 29 days (D).

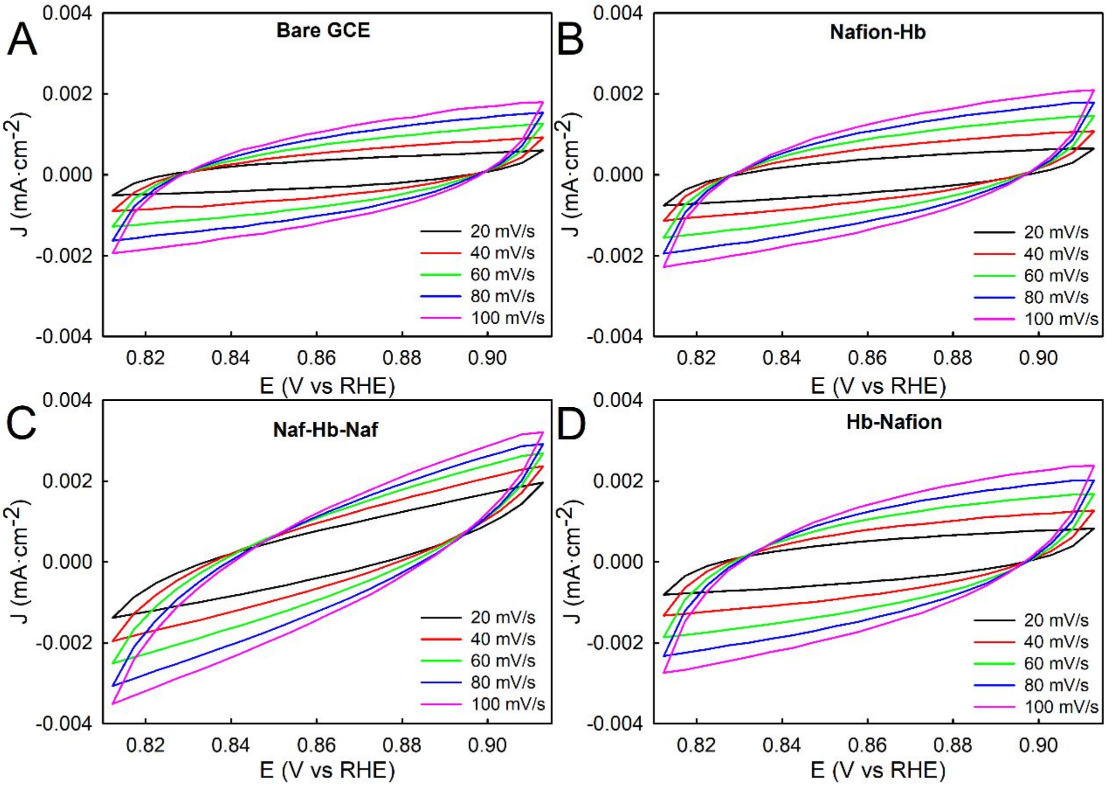

Figure S5. Cyclic voltametric (CV) curves at different scan rates obtained in a non-faradaic region and in nitrogen-saturated 0.1 M PBS (pH 7.4) electrolyte solution for (A) bare GCE, (B) Nafion-Hb modified GCE, (C) Nafion-Hb-Nafion modified GCE, and (D) Hb-Nafion modified GCE.

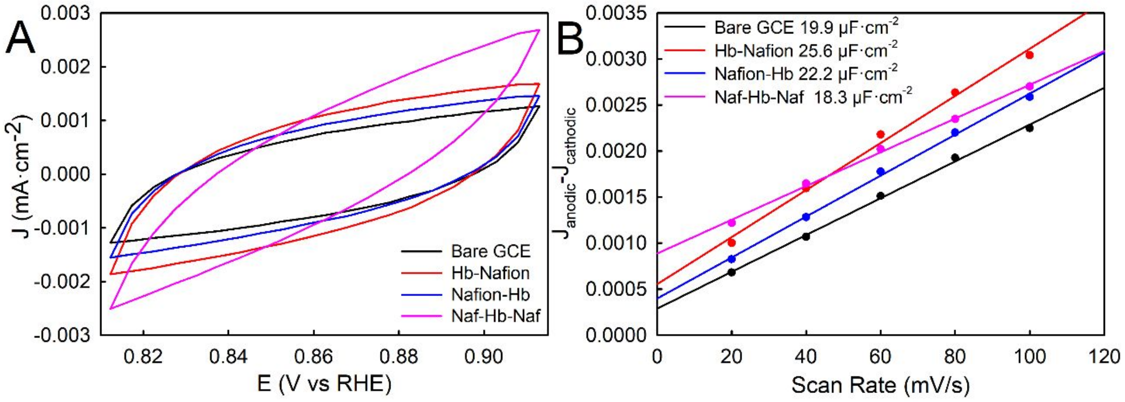

Figure S6. (A) CV curves at 60 mV·s^{-1} and (B) corresponding plots of the difference between anodic and cathodic current densities as a function of the scan rate for the same samples, including bare GCE as a control, obtained through **Figure S5**.

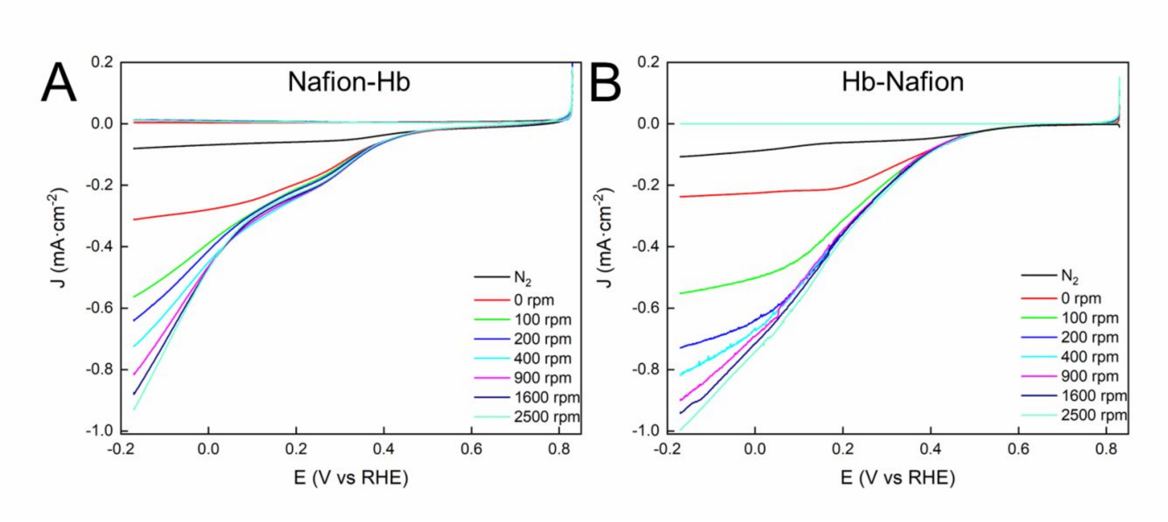

Figure S7. Linear sweep voltametric (LSV) curves of the different electrode modifications, Nafion-Hb (A) and Hb-Nafion (B), obtained using a rotating ring-disk electrode (RRDE) in O_2 and N_2–saturated 0.3M PBS. Rotation rate values are indicated in each graph.

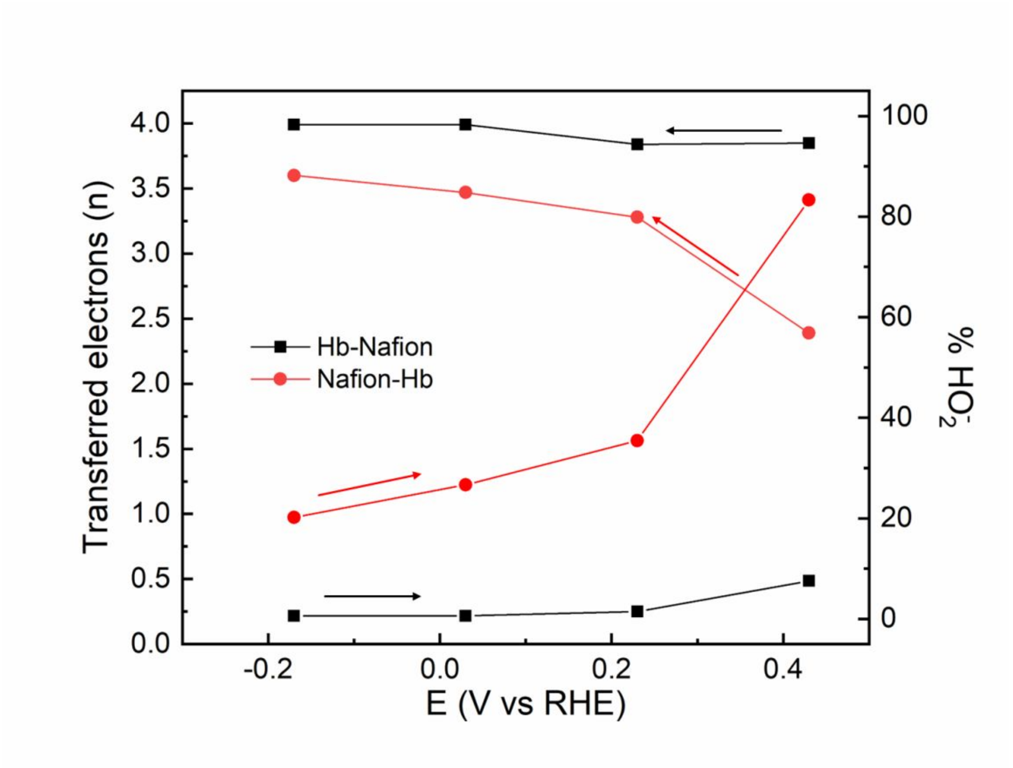

Figure S8. Dependence of the electron transfer number and the HO_2^- values with electrode potential 1.4 V vs RHE, and at rotation rate of 1600 rpm from data in **Figure S7** using Equations (S1) and (S2).

$$n = \frac{4 \cdot I_{disk}}{I_{disk} + \left(\frac{I_{ring}}{N}\right)} \qquad \text{Equation S1}$$

$$HO_2^-(\%) = 200 \frac{\frac{I_{ring}}{N}}{I_{disk} + \left(\frac{I_{ring}}{N}\right)} \qquad \text{Equation S2}$$

where I_{disk} is the disk current, I_{ring} is the ring current, and N is the geometric factor of the RRDE, more often denoted as the current collection efficiency of the Au ring, which was determined to be 42.4.[S1,S2]

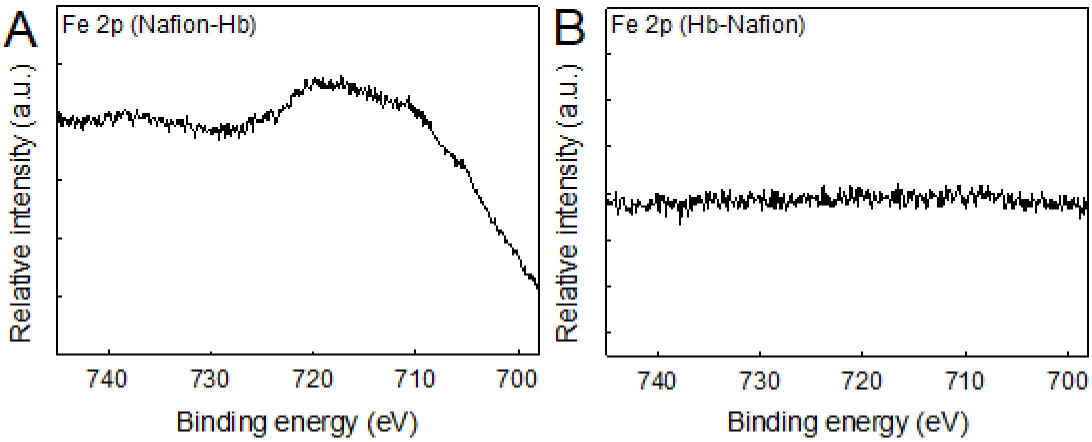

Figure S9. High-resolution XPS spectra of Fe 2p for GDL modified with Nafion-Hb (A) and with Hb-Nafion (B).

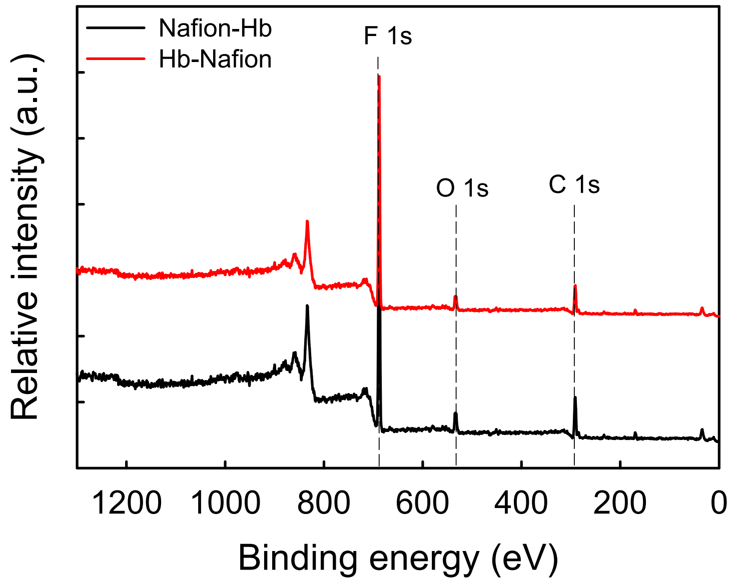

Figure S10. Overall XPS survey spectrum of Nafion-Hb and Hb-Nafion electrode modifications.

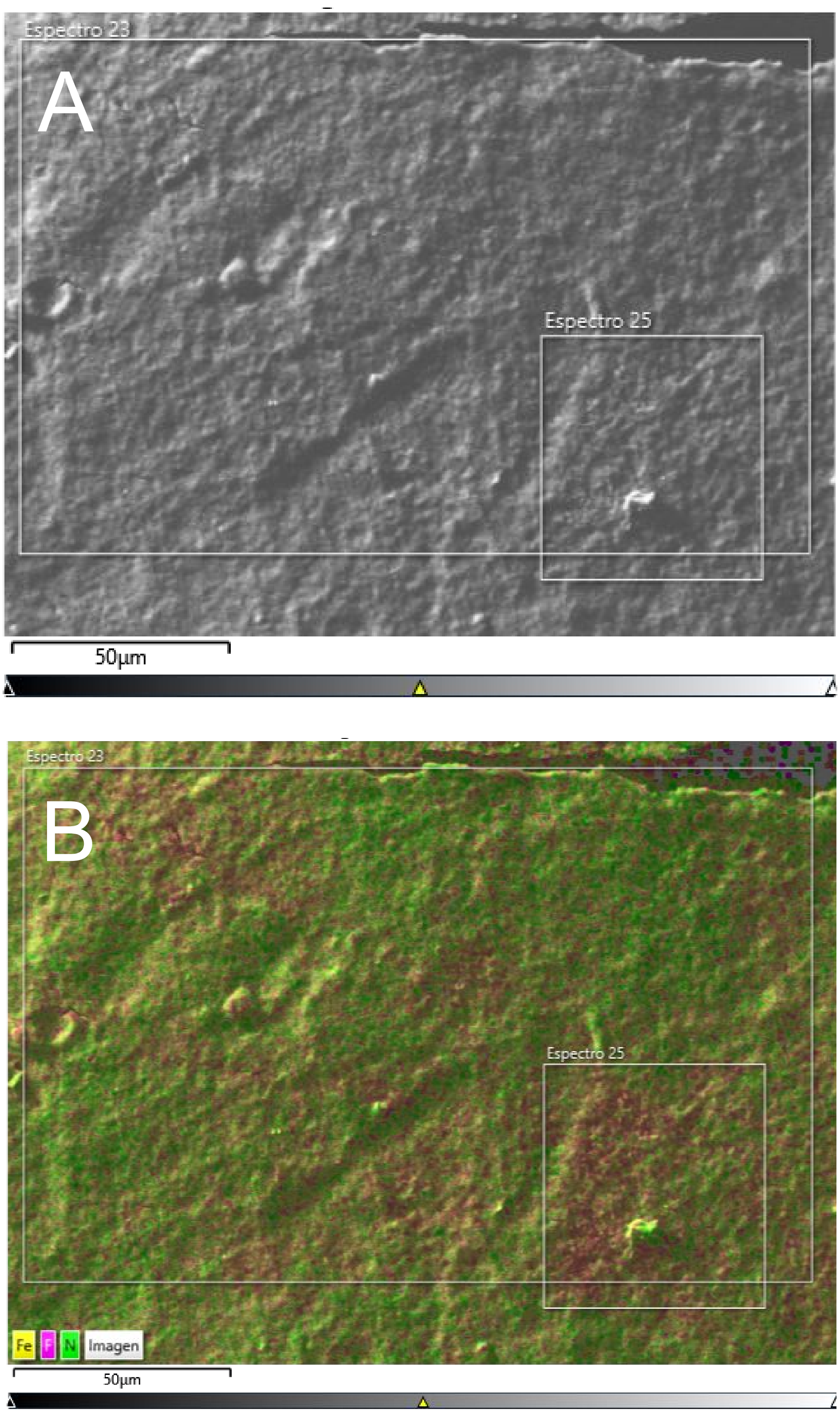

Figure S11. SEM image of a GDL modified with Nafion-Hb (A), and its corresponding overlap of the EDX mapping of the main chemical elements (B).

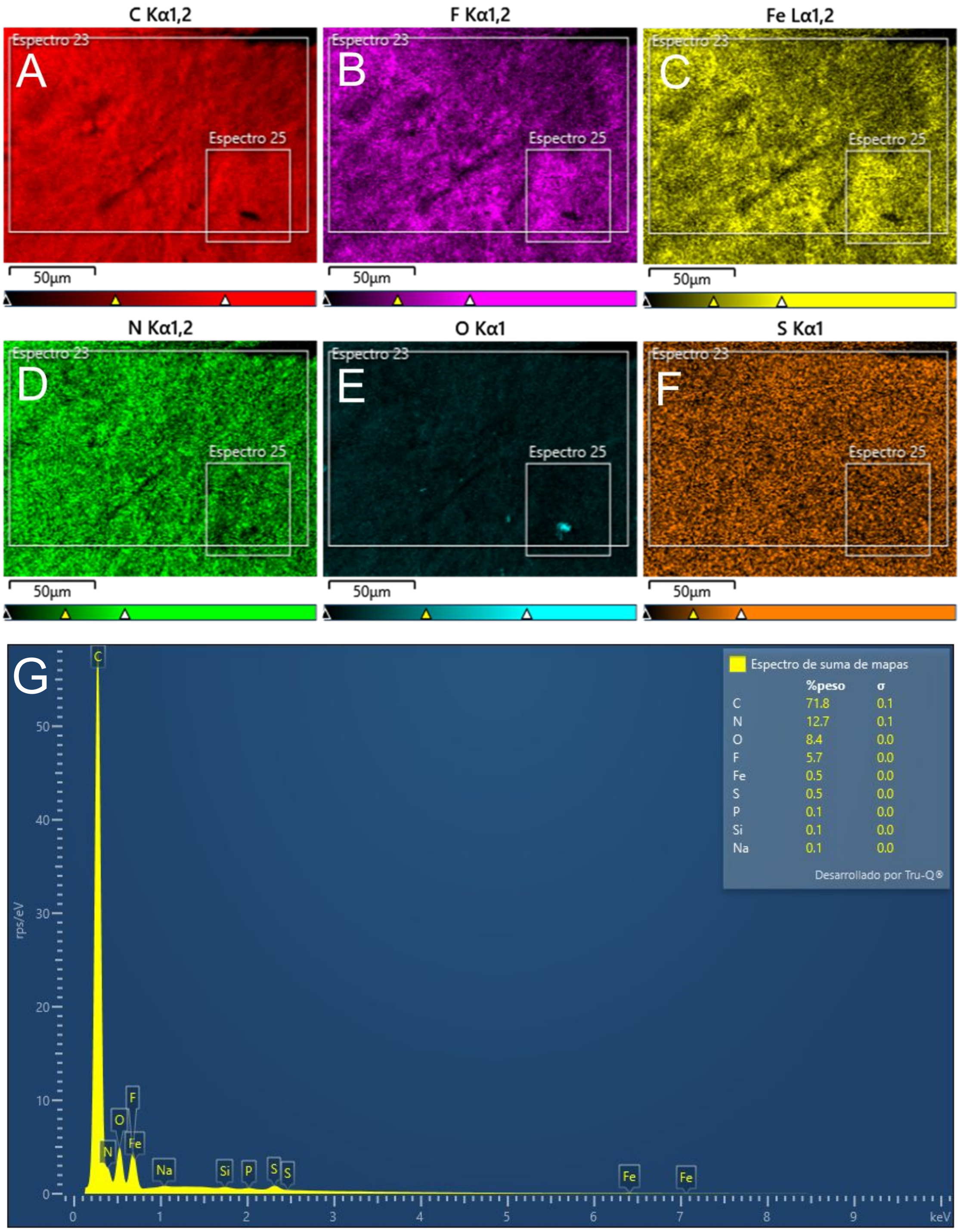

Figure S12. EDX mapping of the chemical elements distribution from **Figure S11**: (A) Carbon (A), Fluorine (B), Iron (C), Nitrogen (D), Oxygen (E), and Sulfur (F). The resulting EDX single spectra (G). Inset: Table containing the weight percentage of various elements.

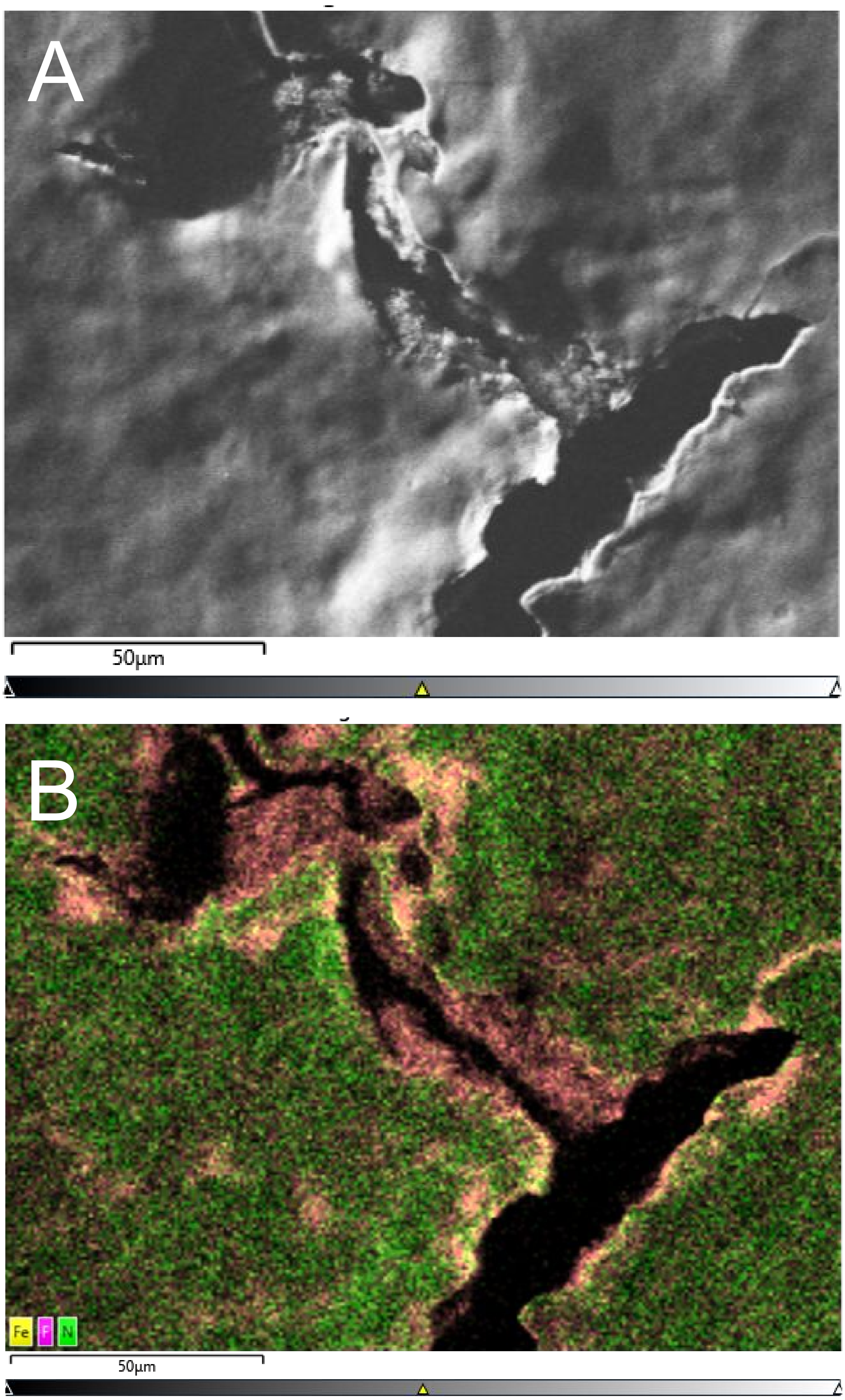

Figure S13. SEM image of a GDL modified with Hb-Nafion (A), and its corresponding overlap of the EDX mapping of the main chemical elements distribution (B).

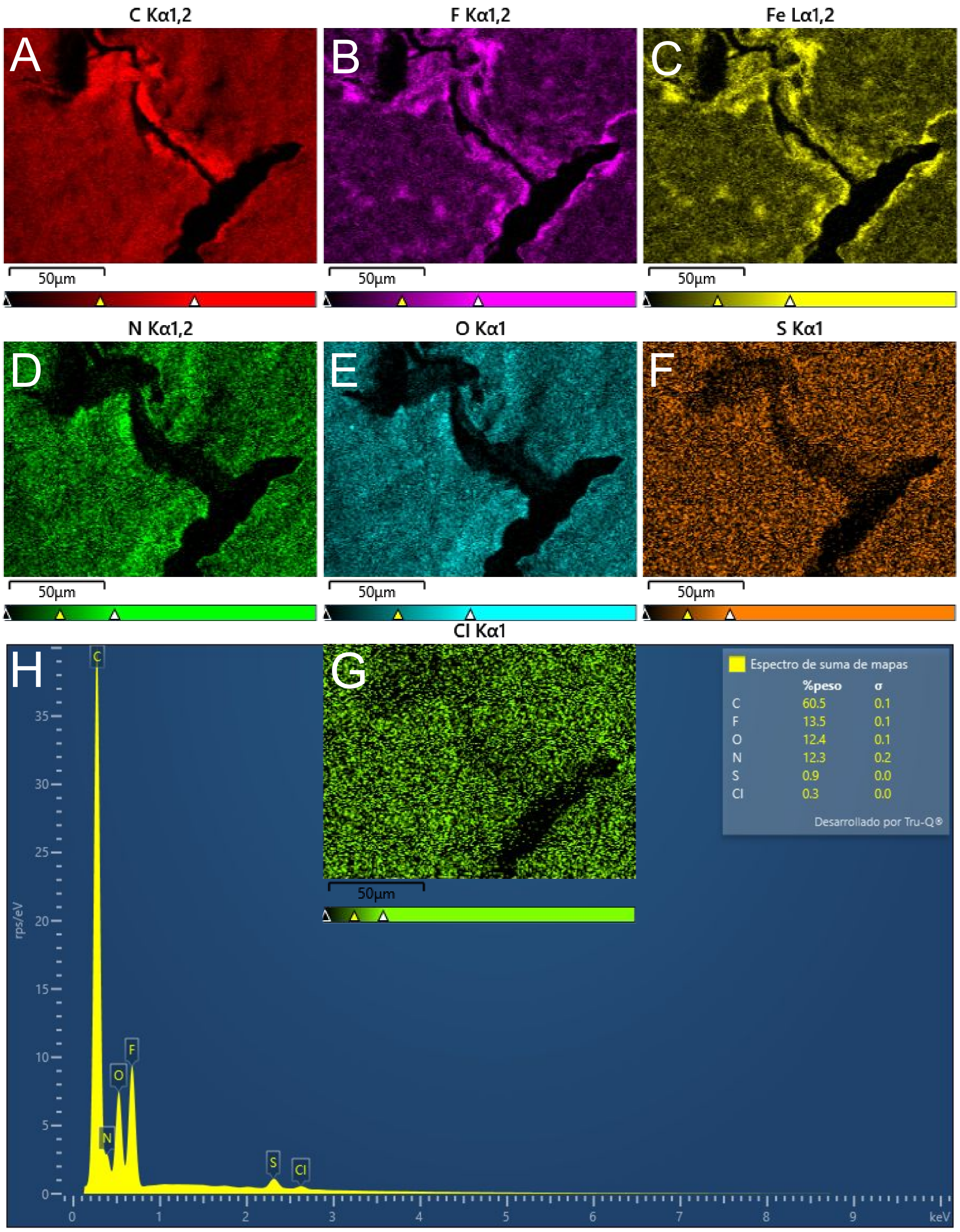

Figure S14. EDX mapping of the chemical elements distribution from **Figure S13**: Carbon (A), Fluorine (B), Iron (C), Nitrogen (D), Oxygen (E), Sulfur (F), and Chlorine (G), (H) The resulting EDX single spectra. Inset: Table containing the weight percentage of various elements.

REFERENCES

(S1) Dalton, F. Historical Origins of the Rotating Ring-Disk Electrode. *Interface Mag.* **2016**, *25*, 50–59.

(S2) Garsany, Y.; Ge, J.; St-Pierre, J.; Rocheleau, R.; Swider-Lyons, K. ORR Measurements Reproducibility Using a RRDE. *ECS Transactions* **2013**, *58*, 1233–1241. https://doi.org/10.1149/05801.1233ecst.
